# Safety and efficacy of the new CryoPop® cryotherapy device for cervical dysplasia in low- and middle-income countries: study protocol for a multicenter open-label non-inferiority clinical trial with historical controls

**DOI:** 10.1186/s13063-021-05802-8

**Published:** 2021-12-13

**Authors:** S. Yogeshkumar, Jean Anderson, Enriquito Lu, Edward Kenyi, Margaret Mensa, Katrina Thaler, Ramalingappa Antartani, Kasturi Donimath, Basavaraj Patil, Santosh Chikaraddi, Shailaja Bidri, Aruna Biradar, Muttappa R. Gudadinni, Laxmikant Lokare, Gayane Yenokyan, Mrutyunjaya B. Bellad, Shivaprasad S. Goudar, Richard Derman, Amit Revankar, Hema Patil, Ramadevi Wani, Ranjit Kangle, Ramesh Y. Chavan, Mahantesh B. Nagmoti, Yogendra M. Kabadi, Purushotham Reddy, Sunita Vernekar, Surekha Hipparagi, Vijayalaxmi Patil, Anita Dalal

**Affiliations:** 1 Jawaharlal Nehru Medical College, KLE Academy of Higher Education and Research (KAHER), Belagavi, Karnataka India; 2 Department of Obstetrics and Gynecology, Johns Hopkins University, School of Medicine, Baltimore, USA; 3 Jhpiego, an Affiliate of Johns Hopkins University, Baltimore, USA; 4 Karnataka Institute of Medical Sciences, Hubballi, Karnataka India; 5 Karnataka Cancer Therapy & Research Institute, Hubballi, Karnataka India; 6 BLDE (Deemed to be University) Shri B. M. Patil Medical College Hospital and Research Centre, Vijayapur, Karnataka India; 7 Johns Hopkins Bloomberg School of Public Health, Baltimore, USA; 8 Department of Obstetrics and Gynecology, Thomas Jefferson University, Philadelphia, USA

**Keywords:** CryoPop®, Cryotherapy, Cervical dysplasia, Low- and middle-income countries

## Abstract

**Background:**

Cervical cancer is the fourth most common cancer in the world, affecting mainly women residing in low- and middle-income countries. Progression from a pre-invasive phase to that of an invasive phase generally takes years and provides a window of opportunity to screen for and treat precancerous lesions.

**Methods:**

This study is being conducted at four sites in north Karnataka, India. Community sensitization activities have been organized in the study areas to create awareness among stakeholders, including elected representatives, physicians, health care workers, and potential participants. Organized community based as well as hospital-based screening is being conducted using visual inspection with acetic acid (VIA). Screen positive women are referred to respective study hospitals for colposcopy and directed biopsy. Participants with confirmed high-grade cervical dysplasia (high-grade squamous intraepithelial lesions or HSIL) who fit all other eligibility criteria will be recruited to the study and will receive cryotherapy using CryoPop®, an innovative new cryotherapy device.

**Discussion:**

There is a need to develop an inexpensive, simple, and effective cryotherapy device for use by frontline health care providers at locations where screening and timely treatment can be given, accelerating access to cervical cancer prevention services and minimizing loss to follow-up of women with precancerous lesions who need treatment.

**Trial registration:**

Clinical Trial Registry - India CTRI/2019/01/017289 ClinicalTrials.Gov number NCT04154644. Registered on November 6, 2019.

## Background

Globally, cervical cancer is the fourth most common cancer in women and was responsible for 311,000 female deaths in 2018. In the next 12 years, the number of women dying annually from this preventable disease is expected to grow by another 100,000 [1]. An overwhelming majority (90%) of cervical cancer deaths occur in low- and middle-income countries (LMICs), with only 5% of global cancer resources allotted to these settings. Investments in scaled up prevention and control measures are far outstripped by the needs [2]. Cervical cancer is relatively unique in that there is a recognizable pre-invasive phase in which progression from oncogenic strains of human papilloma virus (HPV) infection—the primary causative agent of cervical cancer—to invasive disease evolves over several years, passing through distinct precancerous phases known as cervical dysplasia or squamous intraepithelial lesions. This prolonged natural history offers an extended window to detect the presence of precancerous lesions which, when promptly treated, prevent progression to invasive cancer [3–5]. In November 2020, the World Health Organization (WHO) launched its global strategy to accelerate the elimination of cervical cancer. The goal to be achieved by year 2030 includes screening 70% of women by 35 years of age (and again by 45 years of age) and treating 90% of those with precancerous changes [6]. Scalable screening and cost-effective treatment technologies will be required to meet this ambitious goal.

The WHO guidelines for screening and treatment of precancerous cervical lesions [7] recommend a screen- and-treat approach to prevent cervical cancer disease progression, with cryotherapy being designated the first choice of treatment for women who present with a positive screening test. With cryotherapy, observational studies have shown a relative risk reduction in cervical cancer of 86% [8]. Cryotherapy using nitrous oxide (N_2_O) or carbon dioxide (CO_2_) to induce cryonecrosis of dysplastic tissues followed by regeneration of normal cervical epithelium is the most common intervention used in LMICs because it is simple to administer and safe enough for competently trained mid-level practitioners such as nurses and midwives to utilize and can be performed without anesthesia or electricity [9].

Cost, reliability, durability, portability, and reparability are all factors that presently limit the scale-up of treatment necessary to match the volume of population-based screening needed in order to achieve a marked decrease in cervical cancer morbidity and mortality. Each cryotherapy unit costs $2000-$7500, resulting in approximately 80% or more of the treatment cost being directly attributed to equipment cost [10]. Additionally, the current technology requires significant amounts of N_2_O or CO_2_ from large gas cylinders which are heavy and costly—the cost to refill a N_2_O tank can be as high as $930 [11].

**Fig. 1:**
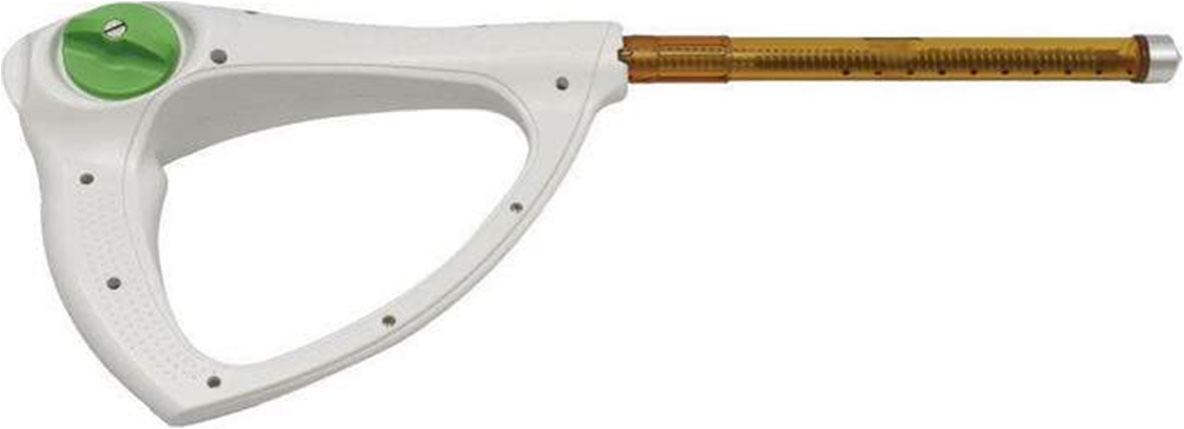
CryoPop® Device (Pregna. Accessed 15 April 2021 https://tinyurl.com/yzrpqwrh)

CryoPop® (see Fig. 1) is a new cryotherapy device developed as a collaboration between Jhpiego, a Johns Hopkins University global health affiliate, and the Johns Hopkins Center for Bioengineering Innovation and Design (CBID) as part of a challenge initiative to address some of the key drawbacks with existing cryotherapy equipment. This device uses the liquid component of CO_2_ to form dry ice as the freezing element. The device is simple, durable, and portable; mechanical problems can easily be repaired on site. As with other cryotherapy devices, it can be utilized by physicians but also task-shared with nurses and midwives. Compared to other cryotherapy equipment, it does not require a tether to a CO_2_ or N_2_O tank; is approximately one-half the cost of standard cryotherapy devices; and is more CO_2_ efficient, using one tenth of the CO_2_ per procedure compared to standard cryosurgery equipment.

The phase 1 of this study (data not shown-paper in progress), completed in the Philippines, was primarily focused on determining the performance characteristics of the CryoPop® as compared to standard cryotherapy equipment. Women with normal cervical cytology scheduled for hysterectomy for benign conditions received cryotherapy 24–48 h before their operation. In a total of 60 women who received cryotherapy with CryoPop® and 20 women who were treated with standard cryotherapy, analysis indicated that CryoPop® was non-inferior to standard cryotherapy in attaining maximum depth of necrosis on the anterior and posterior cervix, the measures used as surrogates for efficacy in published literature [12, 13]. The last 40 women who received cryotherapy with CryoPop® had the length of the freeze-thaw-freeze cycle vary with factor analysis suggesting that longer thaw and longer second freeze were associated with greater depth of necrosis measurements.

This paper presents the CryoPop® Phase 2 clinical trial protocol for the multicenter open-label non-inferiority clinical trial with the objective to compare the safety and efficacy of the CryoPop® device in LMICs with historical controls when treating women with histopathologically confirmed high-grade squamous intraepithelial lesions (HSIL). The original protocol (Protocol version 1.0, January 15, 2019) was approved in 2019 for the Philippines and a current version (Protocol version 2.0, November 20, 2020) reflects approval for India in 2020, with two primary health centers added for colposcopy. All protocol amendments were approved by the ethics boards and any future modifications will be communicated to all relevant parties.

## Methods/design

### Study setting

The study is conducted through a grant from the U.S. National Institutes of Health (NIH) National Cancer Institute as part of the Affordable Cancer Technologies (ACT) Program awarded to the Johns Hopkins University (JHU) and implemented by the Jhpiego Corporation, a global affiliate of Johns Hopkins University, with a sub-contract to the KLE Academy of Higher Education and Research (KAHER) at the Jawaharlal Nehru Medical College (JNMC) in Belagavi, Karnataka, India. KAHER has a longstanding history of successful implementation of NIH-funded research. They have identified additional sites for study implementation in two other cities, Hubballi and Vijayapur. A list of study sites may be obtained by contacting India research coordinator (YK). Strengths of Indian and specifically KLE-affiliated sites include a high prevalence and incidence of cervical cancer; low HIV prevalence (HIV infection associated with greater likelihood of ineligibility for cryotherapy), experience with visual inspection with acetic acid (VIA) for cervical screening and with cryotherapy, availability of experienced colposcopists and pathologists, strong systems in place for community sensitization and mobilization, and good infrastructure and clinical capacity for suspect cancer cases and procedure complications.

**Fig. 2:**
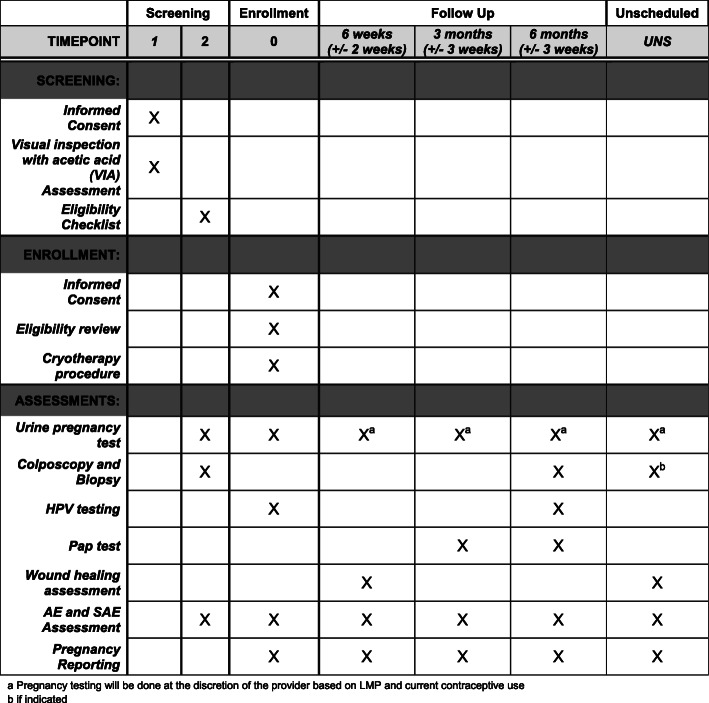
CryoPop® study schedule of enrollment, intervention, and assessments

The study schedule of enrollment, intervention, and assessments is shown in Fig. 2.

### Participants recruitment and selection criteria

Community sensitization/awareness sessions by Accredited Social Health Activists (ASHA) and important stakeholders will be conducted in study areas of Belagavi, Hubballi, and Vijayapur. Following the community awareness sessions, screening camps will be conducted at the community level at primary and urban health centers in Belagavi, Hubballi, and Vijayapur. After informed consent is obtained, visual inspection of the cervix with acetic acid (VIA) will be offered and women who are VIA positive will be referred to the JNMC Karnataka Institute of Medical Sciences (KIMS), The Karnataka Cancer Therapy and Research Institute (KCTRI), or Shri B. M. Patil Medical College, Hospital and Research Centre to undergo colposcopy and biopsy. To meet the required sample size, it is estimated that screening approximately 10,000 women across all the four sites is required as the prevalence of HSIL is reported to be less than one percent in India [14].

At the time of colposcopy, VIA-positive patients will be counseled about possible treatment options available, pending biopsy results, and details of the research study will be introduced.

Those interested in learning more about the study or interested in participating will be referred to a study team member who will meet with them in person for further discussion.

Posters and/or flyers outlining the study in the local language and in English will also be posted in appropriate areas in selected hospitals and health centers, so women who are interested in learning more about the study will have information as to how to contact a staff member. Women who continue to express an interest in the study will also be given the opportunity to meet privately with a study team member and have her questions addressed. Only women who have histologically confirmed HSIL will meet eligibility requirements for the study and will be presented with the consent form.

The inclusion criteria for the study are women between 30 and 49 years, with HSIL of the cervix (cervical intraepithelial neoplasia 2/3) confirmed on histology, eligible for cryotherapy based on size of lesion (occupies < 75% of cervix) and fully visible on colposcopy or VIA and willing to provide consent. Exclusion criteria include the following: menopausal status, have history of hysterectomy, known HIV infection or active cervical infections, pregnancy, or those with lesions ineligible for cryotherapy.

### Consent process

Women who express interest in the study will be formally screened for eligibility using an inclusion/exclusion checklist and consented for the study prior to any research related interventions. The informed consent form approved by the JHU Institutional Review Board (IRB) was translated from English into Hindi, Marathi, and Kannada (English version is included in the appendix). Informed consent will be obtained by a trained and approved study team member who has successfully completed institutionally required certification in the ethical conduct of human subjects’ research. Understanding of the consent will be assessed using a specially designed consent assessment form. If a woman cannot read, the consent form will be read to her either by a member of the study team or a literate witness of her choosing. For those women who cannot write, they may use a thumbprint to sign the consent form. The consent form will then be signed by the witness and the study team member.

### Study intervention

Once enrolled, participants will be scheduled to have a cryotherapy procedure performed with CryoPop®. It is currently standard procedure for cryotherapy to be scheduled within 1 month of a biopsy to confirm the presence of a HSIL. An HPV test and point-of-care urine pregnancy test will also be performed at the time of the procedure. Cryotherapy with CryoPop® will be performed by a skilled clinician previously trained on using this device. Based on findings during the phase 1 of this study, the freeze-thaw-freeze cycle was altered from the standard time of 3 min, 5 min, 3 min to 1-min freeze, 7-min thaw, and 3-min freeze. During the procedure, performance of the CryoPop® will be monitored to ensure the device functions as intended. After the procedure, participants will be instructed to return for a follow-up visit 6 (± 2) weeks after cryotherapy in order to assess healing and interval complications. The woman will be counseled to return at any time prior to this if she has signs or symptoms of complications. Subsequent follow-up visits will occur at three and six months (± 3 weeks). Pap smears will be performed at 3 months and Pap smears, as well as HPV testing, colposcopy, and biopsy of any abnormal areas will occur at the 6-month visit. At follow-up visits, pregnancy testing may be done by a provider based on last menstrual period (LMP) and current contraceptive use. After the trial and post-procedure follow-up are completed, longer term follow-up will be recommended based on current national guidelines for follow-up after treatment for HSIL.

Participants who fail to come to scheduled visits will be contacted by phone or by home visits by the ASHAs to identify concerns or difficulties with follow-up and to assist in addressing these problems. Participants who discontinue participation in the study after undergoing the intervention may be evaluated based on screening, baseline information, and outcomes to the follow-up point reached. Any ancillary care or interventions needed during the study period will be allowed.

### Primary and secondary outcomes

The primary variables of interest are efficacy, safety, healing process, and acceptability. For efficacy, the primary outcome will be the proportion of pap smears and biopsies (if performed) that are negative (have no dysplasia or cancer) at 6 months. It is hypothesized that > 85% of women will have negative Pap smears and colposcopy/biopsy at the 6-month follow-up visit; Pap smears and cervical biopsies will be read by qualified pathologists and will be graded according to accepted and standardized cytologic and histologic criteria.

If there is discordance between the Pap smear report and biopsy readings, the reading with the greater abnormality will have priority as an endpoint. All cytology specimens will be independently read by two nationally and internationally board-certified pathologists whose sub-specialties and work experiences include gynecologic pathology. The JHU pathologist will act as the tie-breaker if there is disagreement. Ten percent of normal cytology readings will also be re-read by the JHU gynecologic pathologist for quality assurance. Histopathologic examinations will be performed on all cervical biopsy samples by the same two pathologists and results will be categorized as normal or negative, low-grade lesions (LSIL), HSIL, invasive cancer, or other. For the purpose of this study, results other than normal or other (not involving dysplasia) will be combined for analysis. If the Pap smear at 3 months shows dysplasia of any grade, colposcopy and biopsy will be performed. A biopsy showing HSIL or invasive cancer will be considered a failure of the procedure and the participant will be removed from the study and standard care arranged and initiated. If the biopsy shows LSIL, the participant will remain in the study and, as per protocol, will have a repeat Pap smear and colposcopy performed at 6 months.

Safety outcomes include the presence of serious adverse events during or within 6 weeks of the procedure (e.g., injury to adjacent structures, infection, excessive bleeding requiring further medical care or transfusion, excessive pain requiring additional medical care, cervical stenosis, etc.)—such anticipated side effects are expected to occur at frequency < 1%. Expected side effects, such as light bleeding or spotting, mild cramping, and vaginal discharge during healing, will also be noted. Type, severity, and timing of side effects and any interventions required will be recorded. Data analysis will include numbers and percentages of any adverse effects, and these will be classified as major or minor, according to pre-existing criteria. Results will be compared to previously noted adverse effects reported in the literature.

Healing at follow-up visit will be assessed subjectively by provider assessment in a dichotomous fashion (completely healed/not completely healed). Performance issues before or during procedures (e.g., adequate fill of applicator with dry ice) will be assessed by the provider using a checklist and scoring system. The provider and participant acceptability will be assessed by the provider and with the use of the participant checklists and score.

### Statistical analysis

The decision was made to perform an open-label uncontrolled clinical trial with comparison to historical controls due to funding constraints for the project. The primary outcome is proportion of women with normal Pap smear (and biopsy if performed) at 6 months post treatment. Sample size was calculated to have at least 80% statistical power to detect non-inferiority of the CryoPop® compared to the historical efficacy of standard cryotherapy devices for the primary outcome at 0.05 one-sided level of statistical significance. For this purpose, a one-sample *Z* test will be used to compare proportion of women with normal Pap smear after using CryoPop® against the previously reported proportion for commercially available devices. It is postulated that approximately 90% of women will have a negative Pap smear at 6 months of follow-up after CryoPop®. Sample size calculations indicate that an enrollment of 82 patients in the CryoPop® arm is required to show a 10-percentage point difference non-inferiority margin. We plan to recruit an additional 10 patients (12%) to account for potential drop-out of participants post-procedure. It was estimated that screening of 10,000 women would be required to identify adequate enrollees.

### Unanticipated problems/adverse events

Adverse events (AEs) will be captured on a case report form (CRF) which will be completed by a trained study team member. All adverse events will be immediately communicated to principal investigator (PI) and site PI. The site PI will make an initial judgment about whether the AE is unanticipated, poses risk or harm to the participants or others, and is related to the study. Final determination regarding which events require submission to the IRB and NIH will be made by the PI upon discussion with the site PI) within 24 h of the event. Any suspected abuse or illegal activity will be communicated immediately to the PI and site PI.

In case of an injury relating to trial participation, free medical management will be given until resolution. In case of trial related injury or death, KLE University’s Jawaharlal Nehru Medical college, Belagavi, will provide financial compensation, determined as per the relevant law and regulations.

### Data collection, management, and analysis

**Fig. 3:**
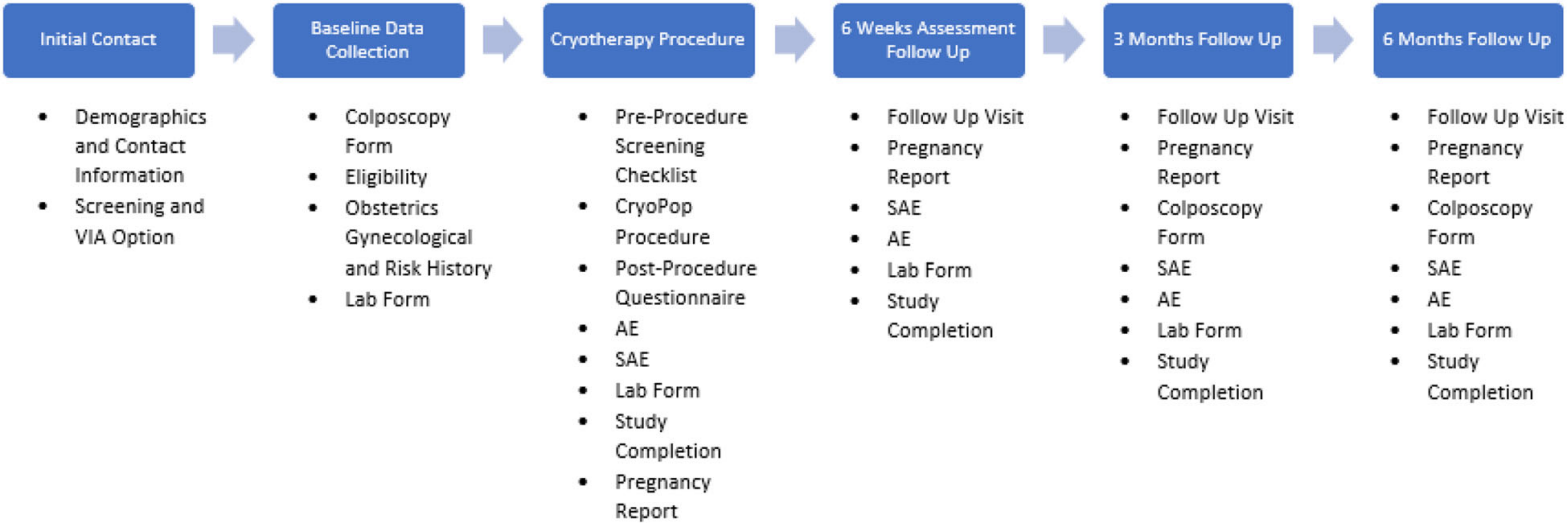
CryoPop® study schedule of case report forms by visits

The study has a data monitoring plan to obtain and review data, to confirm the accuracy and completeness of the data collected, including informed consent forms, source documentation, and CRFs, and to verify that the correct data is entered into REDCap. All processes have specific standard operating procedures (SOPs) to harmonize the processes across all sites and within study teams. All study personnel received a 2-day remote training at the initiation of the study to review study protocol, SOP, CRFs, and overall ethical conduct of the study and management of data. A subsequent review of VIA procedures and interpretation was conducted by the Indian PI. A schedule of CRFs by visit is shown in Fig. 3.

Personal identifiable information (PII) will be collected in hard copy, and all participants will be issued a unique study ID for use on CRFs. All data with PII will be stored separately from CRFs and data with study IDs. Data will be stored in a locked cabinet in a locked room in the study office and accessible only to trained study staff. Data will be entered in REDCap using a secure line. All CRFs and data forms will be stored for 5 years after the completion of the study and then disposed of appropriately per institutional policy.

All de-identification will be performed by KLE University’s JNMC Data Manager, as the Indian Council of Medical Research (ICMR) mandates that India’s site PI retains full ownership of the data.

Some de-identification methods include removing all tagged identifier fields, hashing record IDs, removing notes/essay box fields and date. No identifiable data will leave India. Supplemental de-identification scripts may be run on the de-identified data provided by the India site prior to publishing.

Since the intervention is at a single point of time, the only modifications potentially needed relate to evidence of inferiority of the CryoPop® procedure compared to historical cryotherapy outcomes, as described above. If that occurs, all stakeholders will be notified and the trial stopped. Participants who underwent treatment with the CryoPop® device will be contacted and appropriate follow-up arranged.

There is provision in the consent form for screening that de-identified data from screening efforts may be shared with other investigators conducting future studies. Further details about data management may be obtained by contacting the PI in the US (JA) or Indian PI (AD).

### Study oversight

The PI and the Indian site PI have overall responsibility for the conduct of the study. There is also a Trial Steering Committee, composed of the PI, Indian site PI, research coordinators for India and the USA, study consultants in cervical cancer prevention and women’s health, and the director of research at KAHER. This group meets on Zoom 1–2 times/month to review screening and recruitment, address problems identified, and review data monitoring findings. The data management team consists of a statistician and two data managers (one based in India) who are responsible for uploading and managing data in REDCap and for providing leadership team with basic statistics and identifying missing or inconsistent data elements.

### Data safety monitoring board

A Data Safety Monitoring Board (DSMB) has been established with three members having expertise in epidemiology, pathology, and clinical gynecology, to monitor the safety of the interventions and the validity and integrity of the data from the clinical study. Additionally, the DSMB will evaluate the pace of recruitment and will make recommendations to the PI regarding the continuation, modification, or termination of the study. There will be an interim analysis after 50% of participants have reached the 3-month follow-up point; the study will be terminated if the CryoPop® arm is found to be inferior to the historical controls.

### Dissemination of results

Dissemination of trial results will be through presentation at stakeholder and scientific meetings and through journal publications. Data sharing will be granted in accordance with the details registered with the Clinical Trial Registry of India (CTRI). Individual participant data that underlie the results reported in specific publication arising out of the trial, after de-identification (text, tables, figures, and appendices) will be shared. Additionally, the study protocol, statistical analysis plan, and informed consent forms will be shared. These files will be viewable by researchers whose proposed use of the data has been approved by the Trial Steering Committee. Data will be available beginning 3 months and ending 5 years following article publication.

### Ethical approvals

This study received ethical approval from the Johns Hopkins University’s Bloomberg School of Public Health IRB in Baltimore, United States of America, and the Institutional Ethics Committee of the collaborative partner in India Jawaharlal Nehru Medical College, KLE Academy of Higher Education and Research. Ethical approvals of Karnataka Institute of Medical Sciences (KIMS), The Karnataka Cancer Therapy and Research Institute (KCTRI), or the Shri B. M. Patil Medical College, Hospital and Research Centre, have been obtained. The clinical trial has been registered both in the USA and in India (ClinicalTrials.Gov number NCT04154644 and Clinical Trial Registry - India CTRI/2019/01/017289).

### Study modifications due COVID-19 pandemic

While the study prepared for initiation in April 2020, all human subject research endeavors were paused due to the COVID-19 pandemic. The PI was required to submit plans for protecting the study team members, research participants, and community members and to redesign data collection procedures to meet prevention requirements, including defining occupancy limits in research spaces, imposing physical distancing measures, providing personal protective equipment, establishing protocols for cleaning research spaces, and training study teams on all these procedures.

The study sites are following the local requirements for social distancing, providing N95 masks, gloves, gowns, face shields, and sanitizers for all staff, screening for COVID symptoms of both site staff and participants, and for providing adequate sanitization of all areas at each site. COVID-19 testing is performed on study staff or participants if they are positive on symptom screening, thermal screening or have known contact with COVID-19 +ve person. Local guidance is followed for contact identification, self-isolation, and return to work plans for exposed staff. Social distancing from participants is observed to the extent feasible. A list of documents generated by the India Ministry of Health to provide guidance is filed within Standard Operating Procedures.

Due to COVID-19 travel restrictions, onsite monitoring of the data cannot currently take place, so necessary monitoring will be completed remotely until travel restrictions are lifted.

## Discussion

The main objective of this trial is to determine whether the CryoPop® device is safe and effective in the treatment of high-grade cervical dysplasia or HSIL. Most cancer screening activities in India are still in their infancy, either conducted on an ad-hoc basis by some Non-Governmental Agencies (NGOs) or in some of the reputed cancer centers. As LMICs move towards achieving WHO target goals for prevention of cervical cancer, a range of treatments options will be needed, including cryotherapy, thermal ablation, and loop electrosurgical excisional procedure (LEEP). Decisions regarding specific treatment modalities adopted will include considerations such as cost of equipment and supplies, need for electricity or anesthesia, ease of use, durability of equipment, and ability to scale up in a variety of provider cadres. CryoPop® is inexpensive, CO_2_ efficient, portable, and can be used by midlevel provider cadres and as such can be a valuable addition to available resources for preventing cervical cancer. It can help ensure a screen and treat approach for cervical precancerous lesions, improving coverage at the community level and reducing loss to follow-up.

Preclinical testing with temperature assessment using ballistic gel with CryoPop® in head-to-head comparison with some currently used cryotherapy devices demonstrated that the average tip temperature achieved with CryoPop® is lower than commercially available devices using CO_2_ with good performance over the entire freeze cycle and with significantly lower variation in temperature. Animal testing with CryoPop® showed that the device produced effective necrosis in live tissue and confirmed that the temperatures produced at the CryoPop® tip are in the appropriate range for effective epithelial layer ablation.

Because this device is not considered substantially different from existing cryotherapy equipment from a regulatory perspective, it is able to be commercially manufactured and marketed without additional data. However, it is hoped that the data on efficacy will strengthen the evidence base on the effectiveness of cryotherapy and more specifically on the utility for use of this new and simple device.

In conclusion, the ultimate goal is to assess the safety and efficacy of an inexpensive, simple, and efficient new cryotherapy device which can be implemented at the frontlines of patient care where screening and immediate treatment can best be given. Accelerating access to cervical cancer prevention services and providing another alternative for India and other LMICs to consider in their work to ultimately eliminate cervical cancer as a leading cause of morbidity and mortality should remain a high priority.

## Trial status

Participant recruitment started in July 2020, after delays due to COVID-19. Anticipated study completion period is October 2021. Trial registration: ClinicalTrials.gov number: NCT04154644; Clinical Trial Registry - India CTRI/2019/01/017289

## Data Availability

Per NIH guidelines, the study PI created accounts with the NIH eRA Commons and Grants.gov to publicly release de-identified data. The JHU Data Archives will also publish a de-identified dataset. The data may also be publicly released via the Indian Council of Medical Research guidelines under the discretion of the site PI.

## Abbreviations

ASHA: Accredited Social Health Activists
AE: Adverse events
CRF: Case report forms
HPV: Human papilloma virus
HSIL: High-grade squamous intraepithelial lesions
LEEP: Loop electrosurgical excision procedure
LMIC: Low- and middle-income country
LMP: Last menstrual period
LSIL: Low-grade squamous intraepithelial lesions
PI: Principal investigator
VIA: Visual inspection with acetic acid
NIH: National Institute of Health
WHO: World Health Organization
